# Supplementary material for: HIVprotI: an integrated web based platform for prediction and design of HIV proteins inhibitors
Source: J Cheminform. 2018 Mar 9;10:12. doi: 10.1186/s13321-018-0266-y (PMC5845081; doi:10.1186/s13321-018-0266-y)
Supplement: Supplementary file 2 — Additional file 2. Source code of HIVProtI web server. [file 13321_2018_266_MOESM2_ESM.zip › HIVprotI_Source-code/hivprotI/weka-3-6-6/doc/weka/classifiers/package-tree.html]

weka.classifiers Class Hierarchy


---


|  |  |  |
| --- | --- | --- |
| **Overview** \| **Package** \| Class \| **Tree** \| **Deprecated** \| **Index** \| **Help** \| **Weka's home** | |  |
| **PREV**   **NEXT** | **FRAMES**    **NO FRAMES**     **All Classes** |  |


---


## Hierarchy For Package weka.classifiers

**Package Hierarchies:**: All Packages

---

## Class Hierarchy

- java.lang.Object
  - weka.classifiers.**BVDecompose** (implements weka.core.OptionHandler, weka.core.RevisionHandler, weka.core.TechnicalInformationHandler)- weka.classifiers.**BVDecomposeSegCVSub** (implements weka.core.OptionHandler, weka.core.RevisionHandler, weka.core.TechnicalInformationHandler)- weka.core.**Check** (implements weka.core.OptionHandler, weka.core.RevisionHandler)
        - weka.core.**CheckScheme**
          - weka.classifiers.**CheckClassifier**
        - weka.classifiers.**CheckSource** (implements weka.core.OptionHandler, weka.core.RevisionHandler)- weka.classifiers.**Classifier** (implements weka.core.CapabilitiesHandler, java.lang.Cloneable, weka.core.OptionHandler, weka.core.RevisionHandler, java.io.Serializable)
            - weka.classifiers.**MultipleClassifiersCombiner**
              - weka.classifiers.**RandomizableMultipleClassifiersCombiner** (implements weka.core.Randomizable)
              - weka.classifiers.**RandomizableClassifier** (implements weka.core.Randomizable)- weka.classifiers.**SingleClassifierEnhancer**
                  - weka.classifiers.**IteratedSingleClassifierEnhancer**
                    - weka.classifiers.**RandomizableIteratedSingleClassifierEnhancer** (implements weka.core.Randomizable)
                    - weka.classifiers.**RandomizableSingleClassifierEnhancer** (implements weka.core.Randomizable)
            - weka.classifiers.**CostMatrix** (implements weka.core.RevisionHandler, java.io.Serializable)- weka.classifiers.**Evaluation** (implements weka.core.RevisionHandler, weka.core.Summarizable)

## Interface Hierarchy

- weka.classifiers.**IntervalEstimator**- weka.classifiers.**IterativeClassifier**- weka.classifiers.**Sourcable**- weka.classifiers.**UpdateableClassifier**

---


|  |  |  |
| --- | --- | --- |
| **Overview** \| **Package** \| Class \| **Tree** \| **Deprecated** \| **Index** \| **Help** \| **Weka's home** | |  |
| **PREV**   **NEXT** | **FRAMES**    **NO FRAMES**     **All Classes** |  |


---
